# A new classification system for bacterial Rieske non-heme iron aromatic ring-hydroxylating oxygenases

**DOI:** 10.1186/1471-2091-9-11

**Published:** 2008-04-03

**Authors:** Ohgew Kweon, Seong-Jae Kim, Songjoon Baek, Jong-Chan Chae, Michael D Adjei, Dong-Heon Baek, Young-Chang Kim, Carl E Cerniglia

**Affiliations:** 1 Microbiology Division, National Center for Toxicological Research/U.S. FDA, Jefferson, AR 72079, USA; 2 Division of Personalized Nutrition & Medicine, National Center for Toxicological Research/U.S. FDA, Jefferson, AR 72079, USA; 3 Biotechnology Center for Agriculture and the Environment, Cook College, Rutgers University, New Brunswick, NJ 08901, USA; 4 Department of Health Norfolk Department of Public Health Bureau of Laboratories, Norfolk, VA 23510, USA; 5 Department of Oral Microbiology and Immunology, School of Dentistry, Dankook University, Chonan 330-714, Republic of Korea; 6 School of Life Science, Chungbuk National University, Cheongju, Chungbuk 361-763, Republic of Korea

## Abstract

**Background:**

Rieske non-heme iron aromatic ring-hydroxylating oxygenases (RHOs) are multi-component enzyme systems that are remarkably diverse in bacteria isolated from diverse habitats. Since the first classification in 1990, there has been a need to devise a new classification scheme for these enzymes because many RHOs have been discovered, which do not belong to any group in the previous classification. Here, we present a scheme for classification of RHOs reflecting new sequence information and interactions between RHO enzyme components.

**Result:**

We have analyzed a total of 130 RHO enzymes in which 25 well-characterized RHO enzymes were used as standards to test our hypothesis for the proposed classification system. From the sequence analysis of electron transport chain (ETC) components of the standard RHOs, we extracted classification keys that reflect not only the phylogenetic affiliation within each component but also relationship among components. Oxygenase components of standard RHOs were phylogenetically classified into 10 groups with the classification keys derived from ETC components. This phylogenetic classification scheme was converted to a new systematic classification consisting of 5 distinct types. The new classification system was statistically examined to justify its stability. Type I represents two-component RHO systems that consist of an oxygenase and an FNR_C_-type reductase. Type II contains other two-component RHO systems that consist of an oxygenase and an FNR_N_-type reductase. Type III represents a group of three-component RHO systems that consist of an oxygenase, a [2Fe-2S]-type ferredoxin and an FNR_N_-type reductase. Type IV represents another three-component systems that consist of oxygenase, [2Fe-2S]-type ferredoxin and GR-type reductase. Type V represents another different three-component systems that consist of an oxygenase, a [3Fe-4S]-type ferredoxin and a GR-type reductase.

**Conclusion:**

The new classification system provides the following features. First, the new classification system analyzes RHO enzymes as a whole. RwithSecond, the new classification system is not static but responds dynamically to the growing pool of RHO enzymes. Third, our classification can be applied reliably to the classification of incomplete RHOs. Fourth, the classification has direct applicability to experimental work. Fifth, the system provides new insights into the evolution of RHO systems based on enzyme interaction.

## Background

Microorganisms play indispensable roles in the degradation and detoxification of polycyclic aromatic hydrocarbons (PAHs) in the environment [1,2]. The initiation of the aerobic microbial degradation of PAHs is an oxidative attack [3,4]. The enzymes that catalyze insertion of molecular oxygen into aromatic benzene rings are termed oxygenases [5]. They require transition metals, such as iron and heme, as catalytic centers. Oxygenases that utilize non-heme Fe(II) are called Rieske-type non-heme iron aromatic ring-hydroxylating oxygenase (RHO) whereas others that use heme are cytochrome P450s [6,7]. The term RHO is used herein to denote the Rieske-type non-heme iron ring-hydroxylating oxygenase.

Although RHOs mostly use NAD(P)H as an electron donor and catalyze the same oxygenation reaction, they are remarkably diverse with respect to their structure [3,4,8]. RHOs are multi-component enzymes of two or three protein components consisting of an electron transport chain (ETC) and an oxygenase. Oxygenase components are either homo- (α_n_) or hetero-oligomers (α_n_β_n_) and in each case, the α subunit, called large subunit, contains two conserved regions, a Rieske [2Fe-2S] center and non-heme mononuclear iron. The α subunits are known to be the catalytic components involved in the transfer of electrons to oxygen molecules. The ETC that transfers reducing equivalents from NAD(P)H to the oxygenase components consists of either a flavoprotein reductase or a flavoprotein reductase and a ferredoxin [3,4]. An interaction between oxygenase and ETC components is required for the enzyme system to transfer electrons from the electron donor to aromatic hydrocarbon electron acceptor. The RHO enzyme system has been extensively studied in many different microorganisms since the initial reaction mostly determines the aromatic substrate for degradation [9-15].

Classification of RHOs is essentially an effort to organize the information into a system that is useful for understanding the relationship between various aspects of sequence, structure, function and evolution. A three-class system (class I, II and III) was initially instituted by Batie et al. [16]. Based on the number of constituent components and the nature of the redox centers, this classification was able to give systematic information about RHOs. We will refer to this approach as "the traditional classification". With the recent tremendous accumulation of new sequence information on RHOs, there is a current need for a new classification strategy that can transform the multitude of complex data into useful organized information. In this regard, computational phylogenetic analysis of molecular sequence was imperative, which we term "the phylogenetic classification". Several challenges have been introduced using this method. Werlen et al. [17] grouped RHOs into four families based on substrate specificities and sequence alignments with associated distance calculations. This classification emphasized the structure-function relationship of the oxygenase component. However, some RHOs appear not to fit in this scheme probably because of the small RHO sample pool which resulted in the partial observation. Nam et al. [18] also proposed a clustering system based on the homology of the amino acid sequences of terminal oxygenase components. This classification system was more inclusive and well reflected the phylogenetic affiliation among RHOs.

In recent years, we characterized 3 oxygenases, NidAB, NidA3B3 and PhtAaAb, from *M. vanbaalenii *PYR-1 involved in the oxidation of aromatic hydrocarbons [9,10,15,19]. Phylogenetic analysis of these oxygenase components, NidA, NidA3 and PhtAa, showed that they clustered with a new group of α subunits found in *Nocardioides*, *Rhodococcus*, *Terrabacter*, *Arthrobacter *and other *Mycobacterium *spp. [20-25]. Interestingly, RHO genes found in this group of bacteria appear to have features in common; genes for ETC components are not always closely positioned with oxygenase genes (genetic discreteness) and limited numbers of ferredoxin and reductase components are shared by multiple oxygenases (numerical imbalance). Interestingly, ferredoxin components, that are compatible with the oxygenase enzymes from this bacterial group, were often identified to be a [3Fe-4S] cluster type [20,22,23]. The [3Fe-4S] type of ferredoxin has been recently introduced as an ETC component for RHO enzyme systems, the other ferredoxin components being the [2Fe-2S] type. In fact, NidAB and NidA3B3 from PYR-1 were also shown to be compatible with the [3Fe-4S]-type ferredoxin, PhtAc and ferredoxin reductase (PhtAd) [19]. However, the classification system proposed by Batie et al. and Nam et al. can not explain these new RHO features, the [3Fe-4S]-type ferredoxin, genetic discreteness of ETC components and the numerical imbalance between oxygenase and ETC component. In addition, considering that RHOs are multi-component enzyme systems, ETC components along with oxygenases components are also necessary for their functional understanding. Therefore, the new classification system was launched with the aim of analyzing the RHO components as a whole. The classification system not only systematically incorporates each component data but also basically reflects the previous "traditional" and "phylogenetic" classification.

## Methods

### Sequences retrieving

The amino acid sequences of RHO enzymes were retrieved by BLAST searches from public databases including NCBI nonredundant (NR) protein database [26]. From several hundreds of RHO enzymes that were recovered, we first ended up with the selection of 130 RHO enzymes. Among them, 25 "standard" RHO enzymes were chosen (Table 1) and used to construct the new classification system. These standard RHO enzymes are all well known with respect to genetics and enzyme functions. We also took sequence identities among RHO enzymes into consideration for the selection of standard RHO samples, in which highly identical RHOs were only once selected, so as for the samples to be a full representative of RHO enzymes. The size of the 25 standard RHOs as well as their quality as an RHO representative were statistically examined. To evaluate the new classification scheme, 38 RHO samples (Table 2) were selected as test enzyme systems. For some of these test enzymes, the genetic information for both the oxygenase and ETC components are available whereas for others only oxygenase information is available. To apply the new classification system, a total of 130 RHO enzymes, which include additional 67 RHO enzymes, were classified.

**Table 1:** Standard RHO enzymes used in this study.

	Oxygenase		Ferredoxin	Reductase		
		
Standard RHO enzyme system	Gene	Prosthetic group	Prosthetic group	Prosthetic group	Structure	Accession number
Carbazole 1,9a-dioxygenase (*P. resinovorans *CA10) [44]	*carAa*	[2Fe-2S]/Fe^2+^	[2Fe-2S]	FAD/[2Fe-2S]	FNR_N_-type	D89064
Phenoxybenzoate dioxygenase (*Alcaligenes *sp. BR60) [45]	*cbaA*	[2Fe-2S]/Fe^2+^	None	FMN/[2Fe-2S]	FNR_C_-type	U18133
Phenoxybenzoate dioxygenase (*P. pseudoalcaligenes *POB310) [46]	*pobA*	[2Fe-2S]/Fe^2+^	None	FMN/[2Fe-2S]	FNR_C_-type	X78823
Phthalate dioxygenase (*B. cepacoa *DBO1) [13]	*ophA2*	[2Fe-2S]/Fe^2+^	None	FMN/[2Fe-2S]	FNR_C_-type	AF095748
*p*-Toluenesulfonate monooxygenase (*C. testosterone *T-2) [47]	*tsaM*	[2Fe-2S]/Fe^2+^	None	FMN/[2Fe-2S]	FNR_C_-type	U32622
Aniline dioxygenase (*Acinetobacter *sp. YAA) [48]	*atdA*	[2Fe-2S]/Fe^2+^	None	FAD/[2Fe-2S]	FNR_C_-type	D86080
Aniline oxygenase (*P. putida *UCC22) [49]	*tdnA1*	[2Fe-2S]/Fe^2+^	None	FAD/[2Fe-2S]	FNR_C_-type	D85415
2-Halobenzoate 1,2-dioxygenase (*P. cepacia *2CBS) [50]	*cbdA*	[2Fe-2S]/Fe^2+^	None	FAD/[2Fe-2S]	FNR_N_-type	X79076
Benzoate 1,2-dioxygenase (*Acinetobacter*. sp. ADP1) [51]	*BenA*	[2Fe-2S]/Fe^2+^	None	FAD/[2Fe-2S]	FNR_N_-type	AF009224
Anthranilate dioxygenase (*Acinetobacter*. sp. ADP1) [51]	*antA*	[2Fe-2S]/Fe^2+^	None	FAD/[2Fe-2S]	FNR_N_-type	AF071556
Phenanthrene dioxygenase (*Nocardioides *sp. KP7) [11]	*phdA*	[2Fe-2S]/Fe^2+^	[3Fe-4S]	FAD	GR-type	AB017795
Phthalate dioxygenase (*Terrabacter *sp. DBF63) [22]	*phtA1*	[2Fe-2S]/Fe^2+^	[3Fe-4S]	FAD	GR-type	AB084235
Phthalate dioxygenase (*A. keyseri *12B) [23]	*phtAa*	[2Fe-2S]/Fe^2+^	[3Fe-4S]	FAD	GR-type	AF331043
Phthalate dioxygenase (*M. vanbaalenii *PYR-1) [10]	*phtAa*	[2Fe-2S]/Fe^2+^	[3Fe-4S]	FAD	GR-type	AY365117
Phthalate dioxygenase (*Rhodococcus *sp. RHA1) [34]	*padAa2*	[2Fe-2S]/Fe^2+^	[3Fe-4S]	FAD	GR-type	AB154537
3,4-Dihydroxyphenanthrene dioxygenase (*A. faecalis *AFK2) [52]	*phnAc*	[2Fe-2S]/Fe^2+^	[2Fe-2S]	FAD/[2Fe-2S]	FNR_N_-type	AB024945
Naphthalene dioxygenase (*Pseudomonas *sp. 9816-4) [12]	*nahAc*	[2Fe-2S]/Fe^2+^	[2Fe-2S]	FAD/[2Fe-2S]	FNR_N_-type	U49496
PAH dioxygenase (*P. putida *OUS82) [53]	*pahAc*	[2Fe-2S]/Fe^2+^	[2Fe-2S]	FAD/[2Fe-2S]	FNR_N_-type	AB004059
Carbazole dioxygenase (*Sphingomonas *sp. CB3) [54]	*carAa*	[2Fe-2S]/Fe^2+^	[2Fe-2S]	FAD	GR-type	AF060489
Dioxin dioxygenase (*Sphingomonas *sp. RW1) [14]	*dxnA1*	[2Fe-2S]/Fe^2+^	[2Fe-2S]	FAD	GR-type	X72850
Biphenyl dioxygenase (*Rhodococcus *sp. RHA1) [55]	*bphA1*	[2Fe-2S]/Fe^2+^	[2Fe-2S]	FAD	GR-type	D32142
Toluene dioxygenase (*P. putida *F1) [56]	*todC1*	[2Fe-2S]/Fe^2+^	[2Fe-2S]	FAD	GR-type	J04996
Biphenyl dioxygenase (*Pseudomonas *sp. KKS102) [57]	*bphA1*	[2Fe-2S]/Fe^2+^	[2Fe-2S]	FAD	GR-type	D17319
Biphenyl 2,3-dioxygenase (*B. xenovorans *LB400) [58]	*bphA*	[2Fe-2S]/Fe^2+^	[2Fe-2S]	FAD	GR-type	M86348
Biphenyl dioxygenase (*P. pseudoalcaligenes *KF707) [59]	*bphA1*	[2Fe-2S]/Fe^2+^	[2Fe-2S]	FAD	GR-type	M83673

**Table 2:** Test RHO enzymes used in this study.

	Oxygenase		Ferredoxin	Reductase		
		
Test RHO enzyme system	Gene	Prosthetic group	Prosthetic group	Prosthetic group	Structure	Accession number
PAH dioxygenase (*M. vanbaalenii *PYR-1) [9]	*orf25*	[2Fe-2S]/Fe^2^	unknown	unknown	unknown	AY365117
	*nidA*	[2Fe-2S]/Fe^2^	unknown	unknown	unknown	AF249301
	*nidA3*	[2Fe-2S]/Fe^2^	unknown	unknown	unknown	DQ028634
PAH dioxygenase (*Mycobacterium *sp. 6PY1) [25]	*pdoA1*	[2Fe-2S]/Fe^2^	unknown	unknown	unknown	AJ494745
PAH dioxygenase (*Mycobacterium *sp. S65) [24]	*pdoA*	[2Fe-2S]/Fe^2^	unknown	unknown	unknown	AF546905
PAH dioxygenase (*Rhodococcus *strain I24) [21]	*nidA*	[2Fe-2S]/Fe^2^	unknown	unknown	unknown	AF121905
PAH dioxygenase (*Pseudomonas abietaniphila *BKME-9) [60]	*ditA1*	[2Fe-2S]/Fe^2^	[3Fe-4S]	unknown	unknown	AF119621
PAH dioxygenase (*N. aromaticivorans *F199) [37]	*xylX*	[2Fe-2S]/Fe^2^	[2Fe-2S]	FAD	GR-type	AF079317
	*bphA1a*	[2Fe-2S]/Fe^2^				
	*bphA1b*	[2Fe-2S]/Fe^2^				
	*bphA1c*	[2Fe-2S]/Fe^2^				
	*bphA1d*	[2Fe-2S]/Fe^2^				
	*bphA1e*	[2Fe-2S]/Fe^2^				
	*bphA1f*	[2Fe-2S]/Fe^2^				
PAH dioxygenase (*Sphingomonas *sp. P2) [36]	*ahdA1a*	[2Fe-2S]/Fe^2^	[2Fe-2S]	FAD	GR-type	AB091693
	*ahdA1b*	[2Fe-2S]/Fe^2^				
	*ahdA1c*	[2Fe-2S]/Fe^2^				
	*ahdA1d*	[2Fe-2S]/Fe^2^				
	*ahdA1e*	[2Fe-2S]/Fe^2^				
	*xylX*	[2Fe-2S]/Fe^2^				
PAH dioxygenase (*Sphingomonas *sp. CHY-1) [42]	*phnA1a*	[2Fe-2S]/Fe^2^	[2Fe-2S]	FAD	GR-type	AJ633551
	*phnA1b*	[2Fe-2S]/Fe^2^				
PAH dioxygenase (*Cycloclasticus *sp. A5) [43]	*phnA1*	[2Fe-2S]/Fe^2^	[2Fe-2S]	FAD	FNR_N_-type	AB102786
Aniline dioxygenase (*D. acidovorans *7N) [61]	*orf7NC*	[2Fe-2S]/Fe^2^	None	FAD/[2Fe-2S]	FNR_C_-type	AB177545
Phthalate dioxygenase (*P. putida*) [62]	*pht3*	[2Fe-2S]/Fe^2^	None	FMN/[2Fe-2S]	FNR_C_-type	D13229
Vanillate O-demethylase oxygenase (*Pseudomonas *sp. HR199) [63]	*vanA*	[2Fe-2S]/Fe^2^	None	FMN/[2Fe-2S]	FNR_C_-type	Y11521
Anthranilate 1,2-dioxygenase (*P. resinovorans *CA10) [64]	*antA*	[2Fe-2S]/Fe^2^	None	FAD/[2Fe-2S]	FNR_N_-type	AB047548
Naphthalene dioxygenase (*Ralstonia *sp. U2) [38]	*nagAc*	[2Fe-2S]/Fe^2^	[2Fe-2S]	FAD/[2Fe-2S]	FNR_N_-type	AF036940
Salicylate 5-hydroxylase (*Ralstonia *sp. U2) [65]	*nagG*	[2Fe-2S]/Fe^2^	[2Fe-2S]	FAD/[2Fe-2S]	FNR_N_-type	AF036940
Salicylate 5-hydroxylase (*P. aeruginosa *JB2) [66]	*hybB*	[2Fe-2S]/Fe^2^	[2Fe-2S]	FAD/[2Fe-2S]	FNR_N_-type	AF087482
Naphthalene dioxygenase (*P. aeruginosa *Pak1) [67]	*pahA3*	[2Fe-2S]/Fe^2^	[2Fe-2S]	FAD/[2Fe-2S]	FNR_N_-type	D84146
Carbazole 1,9a-dioxygenase (*Pseudomonas *sp. XLDN4-9)	*carAa*	[2Fe-2S]/Fe^2^	[2Fe-2S]	FAD/[2Fe-2S]	FNR_N_-type	DQ060076
PAH dioxygenase (*R. erythropolis *TA421) [68]	*bphA1*	[2Fe-2S]/Fe^2^	[2Fe-2S]	FAD	GR-type	D88021
Chllorobenzene dioxygenase (*Pseudomonas *sp. P51) [17]	*tcbAa*	[2Fe-2S]/Fe^2^	[2Fe-2S]	FAD	GR-type	U15298
Cumene dioxygenase (*P. fluorescens *IP01) [69]	*cumA1*	[2Fe-2S]/Fe^2^	[2Fe-2S]	FAD	GR-type	D37828
*p*-Cumate dioxygenase (*R. palustris *No.7) [70]	*psbAc*	[2Fe-2S]/Fe^2^	[2Fe-2S]	FAD	GR-type	AB022919
*p*-Cumate dioxygenase (*P. putida *F1) [71]	*cmtAb*	[2Fe-2S]/Fe^2^	[2Fe-2S]	FAD	GR-type	U24215
Anthranilate 1,2-dioxygenase (*B. cepacoa *DBO1) [72]	*andAc*	[2Fe-2S]/Fe^2^	[2Fe-2S]	FAD	GR-type	AY223539

### Sequence analysis

ClustalX [27] was used to obtain multiple alignments followed by distance calculations for each RHO component. Pairwise and multiple alignments were carried out with the default parameters. The weight matrix, Gonnet 250, was used to obtain the pairwise distance (PD) matrix. Alignments were visualized and examined using GENEDOC [28]. Phylogenetic trees were constructed by NJ method [29] from distance data and visualized and manipulated with TREEVIEW 1.6.6 [30]. Tree was also generated using iTOL: Interactive Tree Of Life, an online phylogenetic tree viewer and Tree Of Life resource [31]. The reliability of the trees obtained was evaluated by 1000 bootstrap replications. The domain structures for reductase components were analyzed using the Conserved Domain (CD) Database of NCBI [32], which compares primary sequences with all of the known domain structures in the databases.

### Statistical analysis

Matlab™ (The Mathworks, Inc.) was used to implement the proposed algorithm and to perform the simulation. The program under Matlab generates random subsets among 43 RHO samples, performs cluster analysis using the pairwise distance (PD) matrices and obtains the PD value which maximizes the classification accuracy. In the program, ClustalX [27] was used to obtain multiple alignments and PD matrices. The total running time spent for the simulation used in this paper is less than an hour and the matlab source code (.m) of the program is available upon request.

## Results

**Figure 1:**
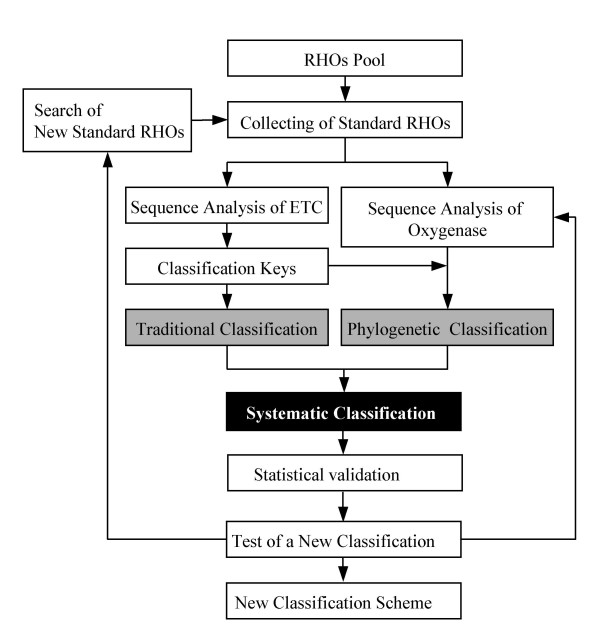
Flowchart for a new classification scheme.

Construction of the new classification scheme was accomplished as shown in the flowchart (Figure 1). Briefly, we first analyzed a total of 130 RHO enzymes in which 25 well-characterized RHO enzymes were selected as standards to test our hypothesis for the proposed classification system (Table 1). From the sequence analysis of ETC components of the standard RHO samples, we extracted the classification keys that reflect the phylogenetic relationship among ETC components, which turned into the grouping criteria in the following classifications. Next, oxygenases were phylogenetically classified with the classification keys obtained from ETC analyses. Finally we constructed the new systematic classification through statistical justification and tested its applicability with 38 RHO samples.

### Step 1: Sampling standard RHOs

Table 1 shows the list of the 25 standard RHO samples whose genetic and functional information were completely available. It includes the members of Batie's three classes and Nam's four groups [16,18] and others that are not mentioned in those classifications. They are phenanthrene dioxygenase of *Nocardioides *sp. KP7 [11] and phthalate dioxygenases of *Terrabacter *sp. DBF63 [33], *Arthrobacter keyseri *12B [23], *M. vanbaalenii *PYR-1 [10] and *Rhodococcus *sp. RHA1 [34]. The ferredoxin components of these RHO enzymes are the [3Fe-4S] type ferredoxin; only [2Fe-2S] type ferredoxin components were analyzed in the previous classification.

### Step 2: Sequence analysis of ETC components from standard RHO samples

In step 2, phylogenetic information was obtained from the amino acid sequences of RHO ETC components, which in turn were converted into classification keys for grouping of oxygenase components for step 3 analyses.

#### Reductase components

**Figure 2:**
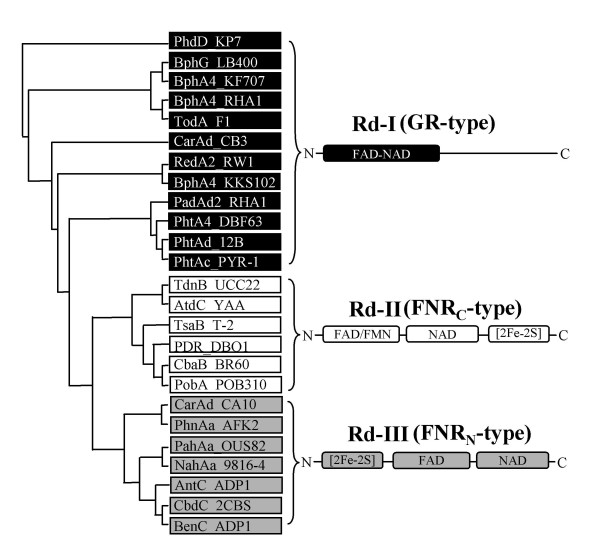
**Grouping of reductase components from 25 standard RHO enzymes with schematic representation of the conserved domain structures**. The names of bacterial strains are indicated after the enzyme names. GR-type, FNR_C_-type, and FNR_N_-type reductases are shown in the boxes with black, gray, and white background, respectively. Designations: FAD-Flavin adenine dinucleotide; NAD-Nicotinamide adenine dinucleotide.

The phenogram with the domain arrangements of reductases is presented in Figure 2. The 25 reductases were divided into three groups, Rd-I, Rd-II and Rd-III, based on the conserved domain arrangements. When grouped with PD value of 0.85, the 25 reductases can also be grouped into the same three groups. Therefore, the PD values obtained by using Gonnet weight matrix were less than 0.85 within each group which has the same arrangement of conserved domains. Group Rd-I consists of glutathione reductase (GR) type reductases that show over 28% amino acid identity to one another, while groups Rd-II and Rd-III include the ferredoxin-NADP^+ ^reductase (FNR) type reductases that show over 15 and 23% amino acid identity within each group, respectively. Groups Rd-II and Rd-III share the same three domains for flavin, NAD and [2Fe-2S] binding, but show different domain arrangements. In group Rd-II, the [2Fe-2S] ferredoxin domains are connected to the C-terminus of NAD domains, but to the N-terminus of flavin-binding domains in group Rd-III. The overall degree of sequence identity between the Group Rd-II and Rd-III is generally no more than 14%. Accordingly, group Rd-I, Rd-II and Rd-III are designated as GR-type, FNR_C_-type and FNR_N_-type reductases, respectively, and were selected as classification keys for the reductase components of RHO enzymes.

#### Ferredoxin Components

**Figure 3:**
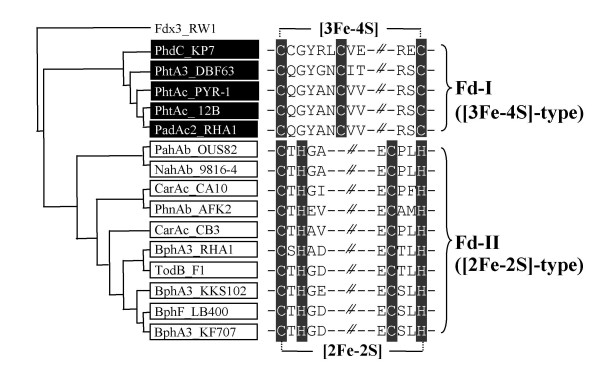
**Grouping of ferredoxin components from standard RHO enzymes with the conserved sequence alignments**. The amino acid residues involved in binding to [2Fe-2S] (CXHX_n_CXXH) and [3Fe-4S] (CX_5_CX_n_C) cluster are indicated by highlighted characters. Fdx3 was used as an outgroup. The names of bacterial strains are indicated after the enzyme names.

Figure 3 shows the result of phylogenetic analysis for the ferredoxin components of standard RHO systems. Conserved amino acid residues were also shown to reveal the classification keys for ferredoxins. For the sequence alignment of ferredoxins, we initially aligned [2Fe-2S]-type and [3Fe-4S]-type ferredoxins separately and identified conserved amino acids. Multiple sequence alignment for the all ferredoxin sequences was then performed, from which we evaluated the validity of alignment. The tree shows that the 16 ferredoxins are divided into two distinct groups based on the type of iron-sulfur clusters, designated group Fd-I and Fd-II. The PD values within each group were less than 0.716. While the overall degree of amino acid sequence identity between groups is no more than 20%, members in each group show over 29% sequence identity to one another. In this analysis, Fdx3, a putidaredoxin-type ferredoxin isolated from *Sphingomonas wittichii *RW1, was used as an outgroup because Fdx3 is phylogenetically unrelated to those of RHO enzymes [35]. The group Fd-I comprises the [3Fe-4S] cluster-containing ferredoxins. Sequence alignments of this group revealed three conserved cysteine residues which serve as ligands for the [3Fe-4S] cluster. Group Fd-II consists of the [2Fe-2S] cluster-containing ferredoxins containing a highly conserved [2Fe-2S]-binding motif, CXHX_n_CX_2_H. Accordingly, group Fd-I and Fd-II are referred as [3Fe-4S]- and [2Fe-2S]-type ferredoxins, respectively, which in turn were selected as classification keys for ferredoxin components of RHOs.

### Step 3: Sequence analysis and grouping of oxygenase components from standard RHO samples

**Figure 4:**
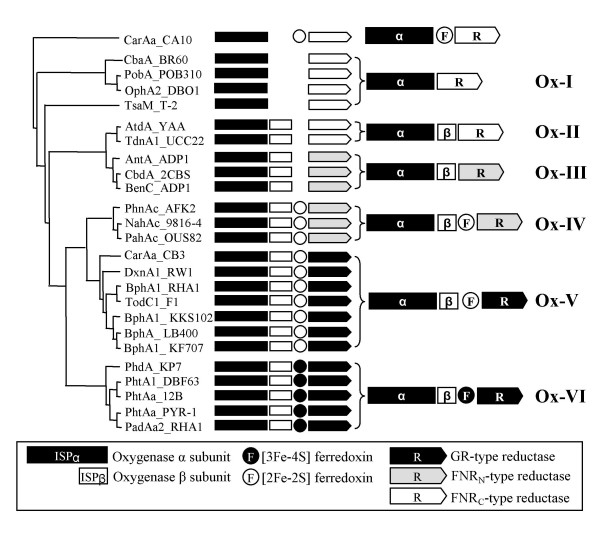
**Phylogenetic clustering of oxygenase components from standard RHO enzymes with regard to the type of their ETC components**. The tree was constructed by the NJ method with CarAa from *P. resinovorans *CA10 as an outgroup.

This step involves integration of the phylogenetic data of oxygenase components with respect to the classification keys obtained from ETC components. Figure 4 shows the dendrogram of 25 oxygenase components of standard RHO samples by the neighbor-joining (NJ) approach with CarAa from *Pseudomonas resinovorans *CA10 as an outgroup. The 25 oxygenase components can be clustered into two groups of homo (α_n_) and hetero (α_n_β_n_) oligomers (Figure 4). The homo-oligomer oxygenases include CbaA from *Alcaligenes *sp. BR60, PobA from *Pseudomonas pseudoalcaligenes *POB310, OphA2 from *Burkholderia cepacia *DBO1, TsaM from *Comamonas testosteroni *T-2 and CarAa from the strain CA10. However, when grouped with the classification keys from the phylogenetic analysis of ETC components, the 25 oxygenases are clustered into six distinct groups, designated Ox-I (an FNR_C_-type reductase and a homo-oligomer oxygenase (α_n_)), Ox-II (an FNR_C_-type reductase and a hetero-oligomer type oxygenase (α_n_β_n_)), Ox-III (an FNR_N_-type reductase and an oxygenase (α_n_β_n_)), Ox-IV (a hetero-oligomer oxygenase (α_n_β_n_), a [2Fe-2S]-type ferredoxin and an FNR_N_-type reductase), Ox-V (a hetero-oligomer oxygenase (α_n_β_n_), a [2Fe-2S]-type ferredoxin and a GR-type reductase) and Ox-VI (a hetero-oligomer oxygenase (α_n_β_n_), a [3Fe-4S]-type ferredoxin and a GR-type reductase) (Figure 4). Groups Ox-I, Ox-II and Ox-III consist of two-component RHO systems, while groups Ox-IV, Ox-V and Ox-VI include three-component RHO systems.

**Figure 5:**
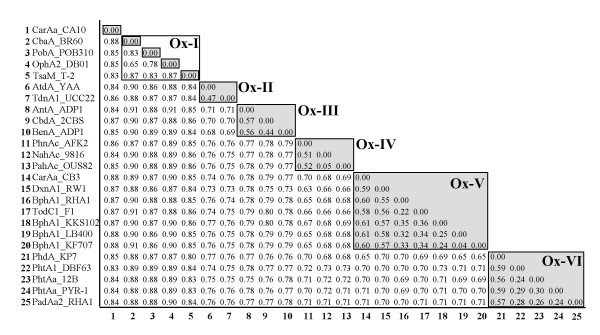
**PD matrix of oxygenase components from standard RHO enzymes by Gonnet 250 weight matrix**. The PD value for grouping in Figure 4 is 0.61. Each group is shown in shadow box with the exception of Ox-I. The group Ox-I can be further divided into four subgroups at the PD value of 0.61.

In Figure 5, the PD values within each group that shares the same classification keys were less than 0.61 with the exception of group Ox-I. This group can be further divided into four subgroups if using 0.61 as a PD value, where 25 oxygenases can be clustered into 10 groups including the outgroup of CarAa from CA10. Therefore, the PD value, 0.61, is a suitable criterion for grouping oxygenases based on our classification keys.

### Step 4: Construction of the new systematic classification

**Figure 6:**
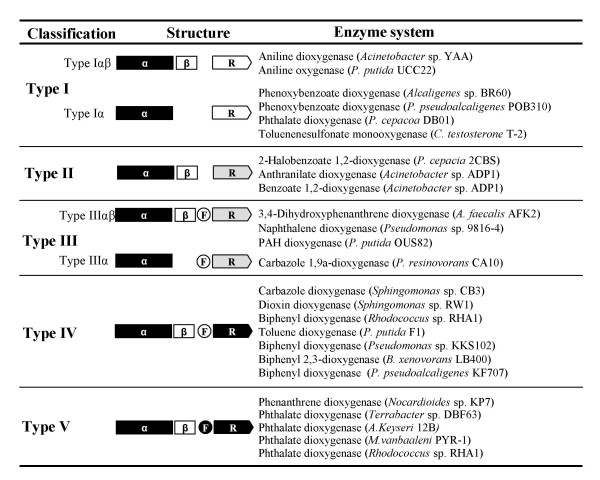
**A systematic classification scheme for standard RHO enzymes**. Schematic diagram of the structures of RHO systems are as in Figure 4.

In this step, the integrated classification data were converted to a systematic classification. As shown in Figure 6, the standard 25 RHO samples can be organized into 5 distinct types from 10 groups which clustered at the PD value of 0.61, designated type I, II, III, IV and V. All the members within each type share the same classification keys of ETC components. Type I and III can be further divided into two subtypes, being designated αβ and α according to the type of oxygenase components in the RHO system; oxygenase components in the subtype αβ and α are hetero-oligomers (α_n_β_n_) and homo-oligomers (α_n_), respectively.

The type I system represents two-component RHOs that consist of an FNR_C_-type reductase and an oxygenase, which is either hetero-oligomer (type Iαβ) or homo-oligomer (type Iα). The RHO systems of group Ox-I and Ox-II belong to the type Iαβ and type Iα, respectively. Type II represents the other two-component systems that consist of an oxygenase and a FNR_N_-type reductase. The RHO enzymes in group Ox-III fall into type II. The well-characterized system for type II is benzoate 1,2-dioxygenase (BenABC) from *Acinetobacter *sp. ADP1. Type III represents three-component systems that consist of an oxygenase, a [2Fe-2S]-type ferredoxin and an FNR_N_-type reductase. All of group Ox-IV belong to type IIIαβ. The carbazole 1,9a-dioxygenase (CarAaAcAd) from *P. resinovorans *CA10 used as an outgroup for reconstructing the dendrogram of oxygenase components was determined to be type IIIα. The type IV systems represent another three-component systems that consist of an oxygenase, a [2Fe-2S]-type ferredoxin and a GR-type reductase. Type IV was shown to be the biggest group for the known RHO enzyme systems. Type V represents another different three-component systems that consist of an oxygenase, a [3Fe-4S]-type ferredoxin and a GR-type reductase. The RHO samples of the group Ox-VI were belonged to type V. The well-known example of the type V enzyme is phenanthrene dioxygenase (PhdABCD) from *Nocardioides *sp. KP7, in which the PhdC was the first [3Fe-4s]-type ferredoxin to be found in RHO enzyme systems.

### Step 5: Statistical justification of the new systematic classification

In this step, we statistically examined the classification scheme; the proposed PD value and the size and quality of the standard RHOs were evaluated using in-house program. Here, the learning accuracy is defined as the ratio of the number of correctly classified observations and the total number of observations.

First, we evaluated which PD value maximizes the classification accuracy for the 25 standard RHO set by the following algorithm:

INPUT: N oxygenases

(1) Select randomly four key oxygenases *a*_2_, *a*_3_, *a*_4 _and *a*_5 _from each categories II, III, IV, V respectively.

(2) At each of the *N*-1 steps *p*_0_, *p*_1_, ..., *p*_n_, the closest two clusters are merged into a single cluster.

(3) Define cut-off distances as *q*_i _= (*p*_*i*-1 _+ *p*_*i*_)/2 for each *i *= 1,..., *n*. For each *q*_*i*_, a cluster is classified as category I, if there is no *a*_2_~*a*_5 _in the cluster. If one or more of *a*_i_'s (*i *= 2, ..., 5) are present in the cluster, the cluster is classified randomly as one of their category. Calculate the learning accuracy and find the cutoff which maximizes the learning accuracy.

**Figure 7:**
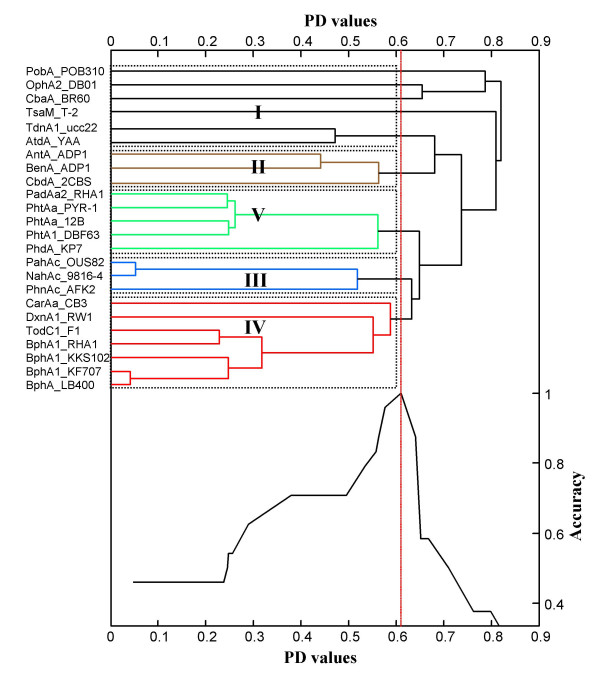
**Accuracy plot and dendrogram of phylogenetic clustering of oxygenase components from standard RHO enzymes using the proposed classification algorithm**. The right vertical axis represents the accuracy of classification based on clustering with the corresponding PD values on the horizontal axis. The labels along the left vertical axis display 25 standard RHO enzymes. At PD value of 0.61 (dashed vertical line), the standard enzymes have the maximum accuracy of 1.0.

Figure 7 is an output data showing the relations among learning accuracy, PD value and RHO classification result deduced from the 25 standard RHOs. This data indicates clearly that the PD value 0.611 satisfies demand for the classification of 25 standard RHO set with a maximum learning accuracy 1.0.

**Figure 8:**
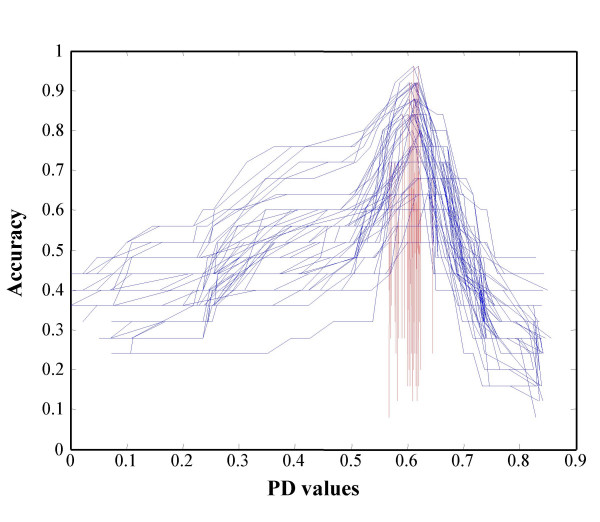
**Accuracy plot using the proposed classification algorithm based on 50 repetitions of 25 randomly selected RHOs**. The vertical axis represents the accuracy and the horizontal axis represents the PD values. Blue solid lines represent the accuracy of classification based on clustering with the corresponding PD values on the horizontal axis. Dashed vertical lines indicate the PD values at which the maximum accuracy is obtained for each repetition.

**Figure 9:**
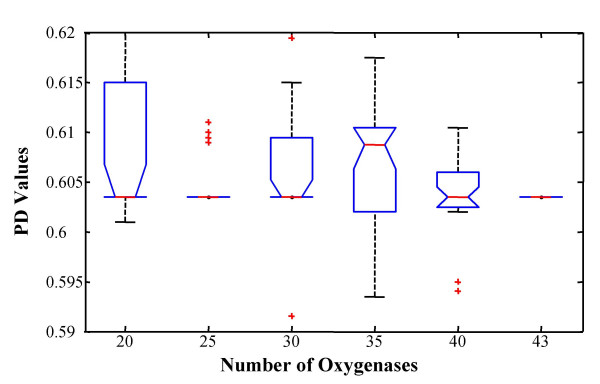
**Box and whisker plot of PD values for randomly selected 20, 25, 30, 35, 40, and 43 oxygenases based on 50 repetitions**. The box has lines at the lower quartile, median, and upper quartile values. Outliers are displayed with a red + sign.

Based on the estimation of PD value, we devised a simulation to determine the suitable set of oxygenases which may minimize the classification error during the prediction. For the purpose of simulation, we used a total of 43 oxygenases which includes 25 standard and 18 test RHO samples. The training sets of 20, 25, 30, 35 and 40 RHOs were randomly selected from a total of 43 oxygenases for 50 times for each training set. Then, we evaluated the PD values which attain the maximum learning accuracy using the above algorithm for each set. Table 3 indicates that the mean deviation of such PD values is close to 0.61 and the values are not much affected by the number of observations, although mean PD values diminishes as the number of oxygenases increases. Figure 8 shows the simulation result based on 50 repetitions for 25 randomly selected RHOs, which indicates that the maximum accuracy is obtained at the mean PD value of 0.619. In Figure 9, it was further shown that the PD values of 25 oxygenases are most stable except for the set of entire 43 oxygenases, although there are some outliers which increase the variance. This simulation test can give us reliability of the 25 standard RHO set and PD value 0.611 for our classification.

**Table 3:** Mean and standard deviation of PD values for randomly selected 20, 25, 30, 35, 40, and 43 oxygenases based on 50 repetitions.

Number of oxygenases	Mean PD value	Standard deviation
20	0.6096	0.0191
25	0.6119	0.0146
30	0.6088	0.0117
35	0.6036	0.0137
40	0.6024	0.0096
43	0.5980	0.0105

### Step 6: Test of the new classification systems

The reliability of the new classification system was evaluated by examining its applicability and usefulness in the classification of 38 RHO enzymes that ranged broadly over the various type of RHO enzymes (Table 2). The selection criteria are as follows. At first, 14 complete RHO enzymes for which both genetic and functional information were well-known were selected. They were chosen for the purpose of verification of the classification systems, which are the same case as those standard RHO samples. Next, the 24 incomplete RHO enzymes were further selected. These incomplete samples include some of the RHO enzymes which have been functionally characterized by gene complementation with compatible source of ETC components and others that have other known equivalent homologs from which ETC information could be deduced.

**Figure 10:**
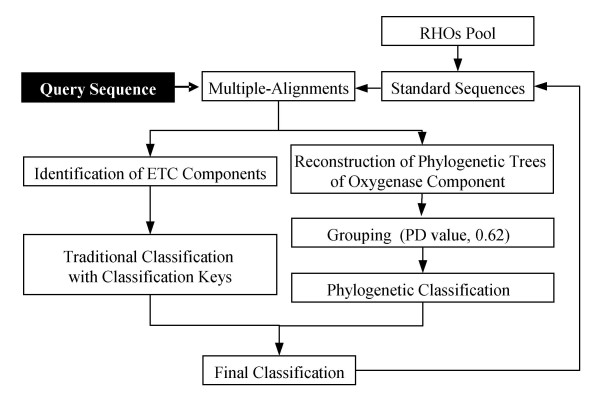
Strategy for query sequence classification used in this study.

Figure 10 shows the strategy used for the classification of test RHO enzymes. Basically, the strategy depends on whether RHOs sequence information is limited. In the case of complete RHO samples, query sequences were first subjected to multiple alignments followed by systematic classification; oxygenase components of RHOs undergo a phylogenetic classification whereas ETC sequences go through traditional classification route which are lastly consolidated into the final classification. In contrast, when analyzing incomplete RHOs, due to the lack of sequence information of the ETC component, only the oxygenase part can be classified according to the phylogenetic analysis. One of the merits of this classification strategy is that it responds dynamically to the growing pool of RHO enzymes. As shown in Figure 10, the output (final classification) of the classification system is returned to increase the standard sequence pool. As the standard RHO pool is growing, the classification gets more stable and objective and could possibly expand its coverage for many other RHO enzymes.

**Figure 11:**
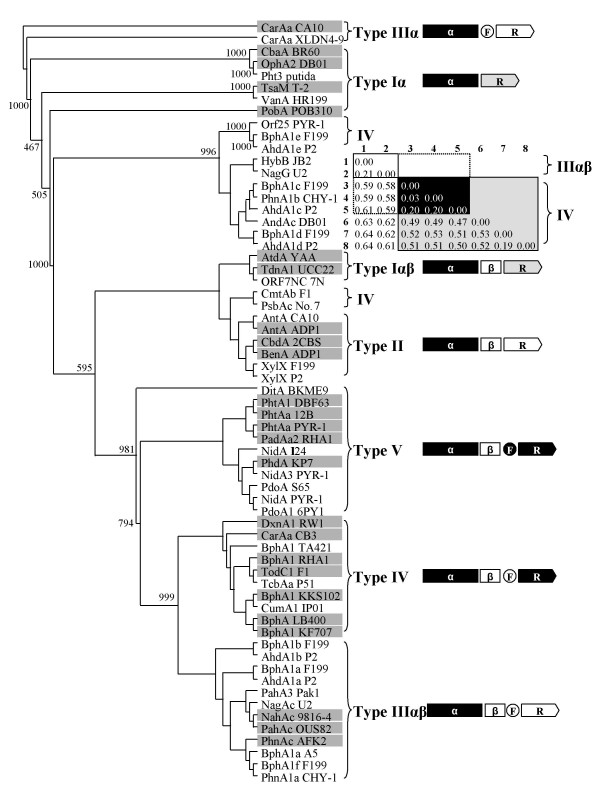
**The resultant tree of the systematic/phylogenetic analysis for 63 RHO enzymes and PD matrix of the type IIIαβ group (HybB and NagG) and type IV group (BphA1c, PhnA1b, AhdA1c, BphA1d, and AhdA1d)**. The standard RHO enzymes are shown in shadow box. Schematic diagram of the structures of RHO systems are as in Figure 4. The tree was constructed by the NJ method with CarAa from *P. resinovorans *CA10 as an outgroup.

Figure 11 shows the result of systematic/phylogenetic analysis for the classification of a total of 63 RHOs which comprised 25 standard and 38 test enzyme samples. This tree has a similar branching structure to that of the standard RHOs samples (Figure 3). It also mirrors well our classification scheme. When analyzing complete test RHO enzymes, the phylogenetic classification results of oxygenase components were in agreement with those of the traditional classification of ETC components. That is to say, ETC sequences of these complete RHO samples were identified by the classification keys to be the same types to which the respective oxygenase components were assigned. For example, both oxygenase (PahA3) and ETC components (PahA1A2) from *P. aeruginosa *Pak1 naphthalene dioxygenase were shown to be typed IIIA by phylogenetic and traditional classification, respectively. It indicates that our systematic classification reflects exactly the relationship/partnership between oxygenase and ETC, which strongly demonstrates that our classification scheme has a potential to classify incomplete RHOs.

The 24 incomplete test RHO samples were all classified and assigned to each type by phylogenetic analysis. For instance, NidA and NidA3 from *M. vanbaalenii *PYR-1 were clustered together with phenanthrene dioxygenase (PhdABCD) from *Nocardioides *sp. KP7 being allocated to type V with a high bootstrap score and stable PD support. Oxygenases from *Novosphingobium aromaticivorans *F199 (BphA1a, BphA1b and BphA1f), *Sphingomonas *sp. P2 (AhdA1a and AhdA1b), *Cycloclasticus *sp. A5 (PhnA1) and *Sphingomonas *sp. CHY-1 (PhnA1a) were clustered into type IIIαβ with high bootstrap and stable PD values. In case of NagG from *Ralstonia *sp. U2, it was classified as type III with the complete RHO, HybB, from *P. aeruginosa *JB2. Another incomplete RHO samples, BphA1c and BphA1d from the strain F199, AhdA1c and AhdA1d from P2 and PhnA1b from CHY-1 were clustered as type IV together with the complete RHO, AndAc, from DBO1. Interestingly, BphA1c from the strain F199, AhdA1c from P2 and PhnA1b from CHY-1 of type IV can also be grouped into type IIIαβ with HybB from JB2 (2-hydroxybenzoate 5-salicylate hydroxylase) based on the PD value of 0.61 (Figure 8). This means that those three type IV RHOs are in the intersection with type IIIαβ. Both XylX oxygenase components from the strain F199 and P2 were grouped into the type II. Notably, ORF25, BphA1e and AhdA1e from the strain PYR-1, F199 and P2, respectively, formed a single distinctive separate cluster with a stable PD value; no standard or complete RHO is clustered together. In this case, although the genetic information of ETC for these three oxygenases are lacking, they were tentatively classified as type IV since BphA1e from the strain P2 was shown to be functionally active when complemented with the ETC component from type IV [36]. In fact, if all the classification keys are combined, we would be able to have 9 possible types for both two- and three-component systems. However, only 5 types were actually applied to classify RHO enzymes and we would need type X (alphabet) for the temporary classification of incomplete RHOs which do not have any standard RHO for comparison.

### Step 7: classification of a total of 130 RHO enzymes

**Figure 12:**
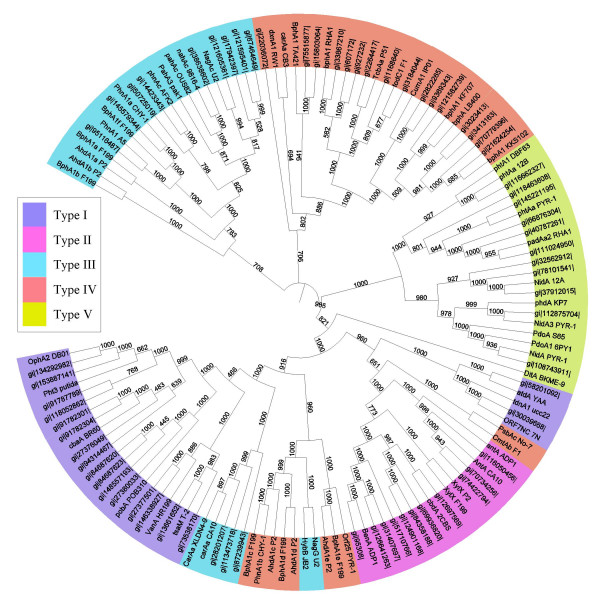
**Classification of a total of 130 RHO enzymes**. Some RHO sequences are provided with gi numbers whose information can be found in NCBI database.

As a final, a total of 130 RHO enzymes, which include 67 new RHO sequences, were classified using our classification system. Figure 12 shows an output based on the new classification system, which has a similar branching structure to that of the test set shown in step 6. Although RHO enzymes seem to have a grouping tendency according to their substrate specificities, the classification clearly indicates that the phylogenetic affiliations of RHO enzymes are determined mainly by their relationship with ETC components. For example, as shown in Type V, PhtAa (phthalate dioxygenase) and NidA3 (fluoranthene dioxygenase) from PYR-1 are grouped together. Although their substrates have been reported to be different, their ETC components have been experimentally shown to be the same as [3Fe-4S]-type ferredoxin and GR-type reductase [9,10,15,19]. In this respect, the results presented in Figure 12 well reflects the phylogenetic affiliations which are integrated with the interactions between RHO enzyme components.

## Discussion

In many cases, RHO oxygenases have not been identified together with ETC components, thus it has been a challenging task to find a cognate ETC partner that can function in cooperation with these "incomplete" oxygenase components [9,35-39]. Since some ETCs are interchangeable with each other and can be often replaced by outside ETC sources and it is the terminal oxygenase component that has the catalytic active site, less emphasis was given to ETC components in Nam's classification. For example, DitA from *P. abietaniphila *BKME-9 and NidA from *Rhodococcus sp*. strain I24 were clustered in group III together with NahAc from *Pseudomonas *sp. NCIB9816-4 and PahAc from *P. putida *OUS82. However, these two oxygenases, DitA and NidA, which lacked ETC information (only ferredoxin for DitA is known), initially formed a group different from that of NahAc and PahAc in the phylogenetic classification route, being classified as type V that represents the three-component systems consisting of an oxygenase, a [3Fe-4S]-type ferredoxin and a GR-type reductase. Whereas the NahAc and PahAc were classified as type III which consists of an oxygenase, a [2Fe-2S]-type ferredoxin and an FNR_N_-type reductase. The discrepancy between these two classification's results comes from ETC information that reflects the relationship among oxygenases. The classification in Nam's scheme was based on pairwise sequence homology of oxygenase components, in which four percentage sets, 0–14, 15–24, 25–34 and 35–99%, were used. In the new classification, however, the grouping criterion for oxygenase components, which is a PD value of 0.62, was objectively determined based on ETC information. This PD value finally determines the two oxygenases, DitA and NidA, differently from the NahAc and PahAc. The results clearly show that information on oxygenases whose ETC components are not available can be used to draw ETC data in our classification system.

This new classification system provides insights into the evolutionary changes and relationships between oxygenase and ETC components. Seven oxygenase components, BphA1 [a-f] and XylX from *N. aromaticivorans *F199 [37] are of particular interest in this context. Although other ETC components are maybe involved, those oxygenases from F199, which were found to be scattered throughout several gene clusters, seem to function with a limited number of ETC components. Two ferredoxins (Rieske- [2Fe-2S]-type BphA3 and plant- [2Fe-2S]-type XylT) and two reductases (GR-type BphA4 and FNR-type XylM) have been identified on the aromatic catabolic plasmid (pNL1) of F199. These oxygenases were classified as type II (XylX), IIIA (BphA1 [a,b,f]) and IV (BphA1 [c-e]) (Figure 8). Although there is no functional data available directly from the strain F199, genetic information with functional evidence have been reported from other sphingomonads which matches with those of F199. For example, sets of genes for an oxygenase from *Sphingobium *sp. P2 (AhdA1 [a-e]) [36], *S. yanoikuyae *B1 (BphA1c and XylX) [40,41] and *Sphingobium *sp. CHY-1 (PhnA1a and PhnA1b) [42] were reported to have the same gene arrangement with 63–97% sequence identity when compared to F199. Consequently, they were classified as the same types with those from F199; type II for XylX (B1), type IIIαβ for AhdA1 [a,b] (P2), PhnA1a (CHY-1) and type IV for BphA1c (B1), AhdA1 [c-e] (P2) and PhnA1b (CHY-1). We found that, without an exception, they were experimentally shown to use type IV ETC (all named as BphA3A4) that consists of [2Fe-2S]-type ferredoxin and GR-type reductase (Figure 6). These ETCs are highly homologous (> 79%) to the BphA3A4 from F199. Therefore, it is reasonable to predict that the 7 oxygenase components (BphA1 [a-f] and XylX) from F199 might be sharing the same type of ETC, BphA3A4. This implies that the limited ETC (BphA3A4) probably makes for strong selective pressure, favoring oxygenase components that can rapidly adapt by increasing their tolerance toward alternative ETC. This adaptation should be accompanied by the change of genetic information, which suggesting that RHO enzymes are probably evolving under the direction of selective pressure derived from ETCs. In fact, although BphA1c from F199 was clustered in type IV, it intersected with type III, the group of 2-hydroxybenzoate 5-salicylate hydroxylase (HybABCD) from *P. aeruginosa *JB2 (Figure 8). It suggests that BphA1c from F199 is probably evolving under the selective pressure derived from limited ETC. The XylX from the strain B1 is also worth noting, XylX belongs to type II, the two-component RHO enzymes, which normally functions with an FNR-type reductase. However, it was unexpectedly shown to be compatible with BphA3A4 [40,41]. Although both ferredoxins from type III and IV are [2Fe-2S]-type, the ferredoxin component (BphA3) from B1 seems phylogenetically inclined to be branched with ferredoxins which can complement oxygenases belong to type IIIαβ. The inclination of the ferredoxin component (BphA3) toward type IIIαβ also can be seen in the RHO enzyme from *Cycloclasticus *sp. A5, of which PhnA1 and PhnA3 components showed high identities over 62% with those (BphA1f and BphA3, respectively) from the strain F199 [43]. In this case, the oxygenase (PhnA1) belongs to type IIIαβ with the ETC components consist of a [2Fe-2S]-type ferredoxin (PhnA3) and an FNR_N_-type reductase (PhnA4). Taken together, the 7 oxygenases from F199, belonging to type II, IIIA and IV, were classified by our system to work with the three different ETC groups, FNR_N_-type reductase, [2Fe-2S]-type ferredoxins/FNR_N_-type reductase and [2Fe-2S]-type ferredoxins/GR-type reductase, respectively. However, all those oxygenases are assumed to function with [2Fe-2S]-type ferredoxin and GR-type reductase. From these observations question may arise about the degree of specificity in recognition between redox partners.

RHO pool is believed to respond dynamically to the environmental transitions such as substrate changes. In addition, relative tolerance between oxygenase and ETC components is also thought to be one of the important selective forces in evolution. In this context, even though little is known as to the role of ferredoxin component under selective pressure, it seems likely to be deeply involved in evolutionary changes. A possible benefit for using ferredoxin comes from its potential to promptly enhance the tolerance toward new redox partners. Cognizant of "the shorter, the faster and more effective", because of the relatively short sequence and simple structure of ferredoxin than that of reductase component, it has an evolutionary merit to adapt rapidly to dynamic environments. Hence, it might be an attractive hypothesis that ferredoxin has been evolutionarily chosen as a buffer between reductase and oxygenase component for rapid adaptation toward selective force. It might also be a strong driving force to affect the direction of evolution from two-component RHO to/toward three-component. Considering this two-way communication flow in RHO system, the adoption of ferredoxin as a new redox partner must have led to the changes in other partners, reductase and oxygenase, in RHO system. In this context, type III RHO seems likely to be a living fossil that has recorded evolutionary changes because they show transitional properties from two-component to three-component system. Two-component systems for type I and II share FNR-type reductase, FNR_C_- and FNR_N_-type, respectively, which consist of three domains (flavin, NAD and [2Fe-2S] binding). On the other hand, three-component systems for type IV and V, except for type III, share a GR-type reductase, which have no [2Fe-2S] cluster (Figure 2 and Figure 6). A [2Fe-2S] cluster of reductases separates the two-component system (type I and II) from the three-component system (type IV and V). It strongly demonstrates that the joining of ferredoxin to the two-component RHO system was probably the start of the type III three-component structure. During evolution, the type III FNR_N_-type reductases might have been gradually changed toward type IV and V GR-type reductases, although it is not sure whether it was recruited from other systems. In some cases, it might also be possible under extreme conditions that type I and II RHO systems evolved directly to type IV (or V) as seen in the XylX from *S. yanoikuyae *B1. Taken together, it may be postulated that RHOs are evolving under selective pressures from type I and II (two-component system) toward type IV and V (three-component), in which this change moves toward efficiency and keeps going continuously under dynamic environments.

## Conclusion

In this study, we have developed a new classification system to classify bacterial Rieske non-heme iron aromatic ring-hydroxylating oxygenases. The new classification system presented in this study not only reflects sequence information but also interactions between RHO enzyme components. The system is characterized by the features that include the following. First, the new classification system analyzes RHO enzymes as a whole, in which information on each RHO components is organized into a system that is useful for the understanding of various aspects with respect to sequence, structure and biochemical function. Second, our new classification system is not static but responds dynamically to the growing pool of RHO enzymes. As standard RHO samples increase, the classification system evolves, which gets more stable and objective and could increase its coverage of many other RHO enzymes. Third, our classification can be applied reliably to the classification of incomplete RHOs. Fourth, the classification has direct applicability to experimental work. Our classification can provide the information about cognate ETC partners for oxygenase catalytic activity. Fifth, the system provides new insights into the evolution of RHO systems based on enzyme interaction.

## Abbreviations

PAH, polycyclic aromatic hydrocarbons; RHO, Rieske-type non-heme iron aromatic ring-hydroxylating oxygenase; ETC, electron transport chain; PD, pairwise distance; NJ, neighbor-joing; CD, conserved domain; NR, nonredundant; GR, glutathione reductase; FNR, ferredoxin-NADP^+^ reductase.

## Authors' contributions

All authors participated in the design of the study and writing of the manuscript. CEC directed the whole research and critically revised the manuscript. All authors read and approved the final manuscript.
